# Correlation of geographical distribution of mycobacteriophages with genomic clusters and genome distance

**DOI:** 10.1186/1471-2105-14-S17-A8

**Published:** 2013-10-22

**Authors:** Miranda L Parrish, Claire A Rinehart

**Affiliations:** 1 Department of Biology, Western Kentucky University, Bowling Green, KY, 42101, USA

## Background

*Mycobacterium smegmatis* is a soil bacterium. Over 448 mycobacteriophages specific for *M. smegmatis* have been sequenced and grouped into clusters of related genomes based on the similarity of their products and genome organization. The phagesdb.org database contains not only the sequence information, but also the geographic locations of the sampling sites for each of these mycobacteriophages. From these data we addressed two questions in this study: one to determine if the mycobacteriophage clusters are randomly distributed geographically, the second to determine the correlation between geographic distance and genetic distance within each cluster.

## Materials and methods

**Figure 1:**
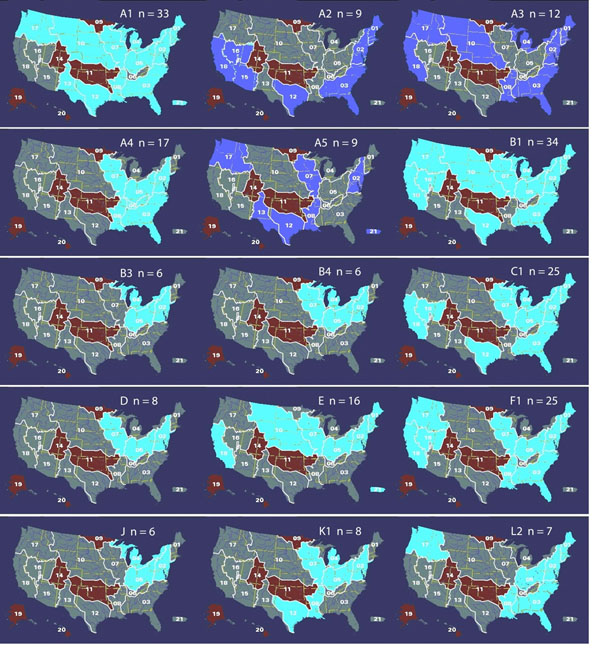
Hydrologic distribution of mycobacteriophages clusters with a sample size, n>5. Cluster maps with cyan regions were not randomly distributed while those with dark blue regions were randomly distributed. The brown regions had no samples in the database and the gray regions did not have cluster representatives in those regions.

Since the geographic sampling was not evenly distributed, the sampling frequency for each geographic and hydrologic [1] region was determined. Samples were drawn at random so that the total number of samples in a cohort was equal to the number of mycobacteriophages contained in a cluster. Each sample was assigned a sampling frequency based on the region from which it was drawn. The sampling probability for the cohort was calculated by multiplying the sampling frequencies for each draw. This was repeated 100,000 times and a sampling probability distribution was generated. The sampling frequency for the actual cluster was also calculated from the geographic frequencies and a probability for the cluster data was determined from the CDF of the sampling distribution. Over half of the clusters showed non-random distribution in both geographic and by hydrologic regions (a = 0.05). Those showing non-random distribution at the hydrologic scale were generally part of adjacent continuous drainage regions (Figure 1).

Geographic distance was determined by the GeoDistance function in Mathematica**^®^** which gives the distance between positions projected onto a reference ellipsoid; heights are ignored. The genetic distance between nucleotide sequences was determined by the DamerauLevenshteinDistance function in Mathematica**^®^** which gives the number of one-element deletions, insertions, substitutions and transpositions required to transform one sequence to the other. A correlation analysis showed very weak or no correlation between geographic distance and genetic distance within clusters.
